# Behavioral health conditions and potentially preventable diabetes-related hospitalizations in the United States: Findings from a national sample of commercial claims data

**DOI:** 10.1371/journal.pone.0212955

**Published:** 2019-02-28

**Authors:** Erica L. Stockbridge, Shlesma Chhetri, Leah E. Polcar, Abiah D. Loethen, Caroline P. Carney

**Affiliations:** 1 Department of Health Behavior & Health Systems, School of Public Health, University of North Texas Health Science Center, Fort Worth, Texas, United States of America; 2 Department of Advanced Health Analytics and Solutions, Magellan Healthcare, Magellan Inc., Scottsdale, Arizona, United States of America; 3 Magellan Rx, Magellan Inc., Scottsdale, Arizona, United States of America; Universita degli Studi di Milano-Bicocca, ITALY

## Abstract

**Objective:**

To characterize the relationship between potentially preventable hospitalizations (PPHs) for diabetes and behavioral health conditions in commercially insured working-age persons with diabetes in the United States.

**Research design and methods:**

We retrospectively analyzed medical and pharmacy claims from services rendered between 2011 and 2013 for 229,039 adults with diabetes. Diabetes PPHs were identified using the Agency for Healthcare Research and Quality’s Prevention Quality Indicators v6.0 logic. We used negative binomial-logit hurdle regression models to explore the adjusted relationships between diabetes PPHs and schizophrenia, bipolar, depression, anxiety, adjustment disorder, alcohol use disorder, and drug use disorder.

**Results:**

A total of 4,521 diabetes PPHs were experienced by 3,246 of the persons in the sample. The 20.83% of persons with one or more behavioral health conditions experienced 43.62% (1,972/4,521; 95% CI 42.18%-45.07%) of all diabetes PPHs, and the 7.14% of persons with more than one diagnosed behavioral health condition experienced 24.77% (1,120/4,521; 95% CI 23.54%-26.05%) of all diabetes PPHs. After adjusting for sociodemographic and physical health covariates, patients with depression, schizophrenia, drug or alcohol use disorders, or multiple behavioral health conditions were at significantly increased risk of experiencing at least one diabetes PPH, while patients with depression, drug use disorder, or multiple behavioral health conditions were at significantly increased risk of experiencing recurring diabetes PPHs over time.

**Conclusions:**

A number of behavioral health conditions are associated with diabetes PPHs, which are often preventable with timely, high-quality outpatient care. The results of this study will enable clinicians, payers, and policy-makers to better focus outpatient care interventions and resources within the population of persons with diabetes.

## Introduction

An estimated 30.3 million people in the United States (US) are living with diabetes [1], a complex chronic disease that is a leading cause of death [2], negatively affects quality of life [3], and is associated with high levels of healthcare utilization [4]. In 2017 in the US, an estimated $237 billion in direct medical expenditures was attributable to diabetes [4]. Persons diagnosed with diabetes have medical costs that are approximately 2.3 times that of persons without diabetes [4], and complications of diabetes represent a significant driver of healthcare costs and utilization in persons with diabetes [5].

Acute and long-term complications of diabetes can often be prevented through ongoing high-quality outpatient medical care [6]. Unfortunately, the quality of outpatient diabetes care may be suboptimal for patients who have comorbid mental health conditions or substance use disorders (collectively termed “behavioral health conditions”). For example, compared to those without substance use disorders, people with comorbid diabetes and alcohol or drug use disorders are less likely to receive recommended hemoglobin A1c (HbA1c) screening [7–9], low-density lipoprotein cholesterol testing (LDL-C) [7–9], microalbuminuria testing [9], foot sensory exams [10], retinal eye exams [7, 8, 10], or nephropathy screening [7], and they are more likely to have poor HbA1c and LDL-C control [7, 8].

The relationship between mental health conditions and the quality of outpatient diabetes treatment is less clear. Depression and anxiety are associated with deficits in diabetes care quality based on many of the measures described previously [8, 9, 11]. However, a literature review of 43 studies found that 65.1% identified no significant association between diabetes care quality and bipolar disorder, schizophrenia, or other psychoses, while 25.6% found lower care quality and 9.3% found higher care quality in persons with these conditions [12]. Differential grouping of clinically diverse behavioral health conditions may explain some of this variation across studies, as studies examining behavioral health conditions individually found that disparities in diabetes care quality varied substantially by condition [8, 11]. Further, people may experience multiple behavioral health conditions, and as the number of different behavioral health conditions increases the likelihood of receiving high-quality diabetes care decreases [8].

While the quality of diabetes care received by persons with both behavioral health conditions and diabetes has been well-studied, the association between behavioral health comorbidities and consequential diabetes-related potentially preventable hospitalizations (PPHs) in individuals with diabetes is relatively under-explored. PPHs, also called prevention quality indicators, are hospitalizations for ambulatory-care sensitive conditions (ACSCs)–conditions that should typically not require hospitalization if patients receive timely, high-quality, community-based primary care or early interventions [13]. Diabetes is one of many ACSCs [13].

Most studies examining PPHs do not focus specifically on diabetes-related PPHs in persons with diabetes. Instead, they use data from general populations [14–19] or veterans [20]. While three studies examined the relationship between comorbid behavioral health conditions and diabetes PPHs in individuals with diabetes, these studies had important limitations. They used non-standard definitions of diabetes PPHs [21], excluded high-prevalence behavioral health conditions (e.g., anxiety, adjustment disorder) [21], did not examine the impact of multiple co-occurring behavioral health conditions [9, 21, 22], or did not investigate whether behavioral health conditions were associated with increased risk of multiple diabetes PPHs over time [9, 21, 22]. Further, these studies focused on people with public insurance [21, 22] or persons residing outside the United States (US) [9], so the relationship between diabetes PPHs and behavioral health conditions has not been explored within the large, heterogeneous population of commercially insured persons in the US with diabetes [23].

The current study examines the relationship between diabetes PPHs and behavioral health conditions in a sizable sample of commercially insured working-age persons with diabetes in the US. We improved on past research a number of ways. In order to more fully characterize the relationship between behavioral health conditions and diabetes-related PPHs, we included a broad range of conditions (i.e., schizophrenia, bipolar, depression and other non-bipolar mood disorders, anxiety, adjustment disorder, alcohol use disorder, and drug use disorder). We also explored the relationship between multiple behavioral health conditions and diabetes PPHs. Additionally, we analyzed individuals’ data over a three-year period to examine recurring diabetes PPHs over time.

Specifically, we hypothesized that each behavioral health condition would be independently associated with an increased likelihood of experiencing at least one diabetes-related PPH as well as multiple diabetes PPHs during a three-year period. Further, we hypothesized that, relative to those with no or only one behavioral health condition, persons with multiple behavioral health conditions would have a greater likelihood of experiencing at least one diabetes-related PPH as well as multiple diabetes PPHs.

## Materials and methods

The North Texas Regional Institutional Review Board at the University of North Texas Health Science Center reviewed and approved this project as exempt category research.

### Data source and analytic sample

We analyzed deidentified medical and pharmacy claims data extracted from the Optum Clinformatics Data Mart [24]. The Clinformatics database contains claims data for approximately 30.6 million commercially insured people in the US, which is roughly 19% of the commercially insured US population. Data reflecting healthcare services and prescriptions for 4 million randomly selected people with continuous commercial health insurance enrollment between 2011 and 2013 were available for analysis. The available data included persons ages 0 to 64 years, and the geographic distribution of these persons approximated the 2010 US population’s distribution into census divisions. Our analyses included a subset of the 4 million people; specifically, we analyzed data for individuals 20 to 64 years of age with diabetes who were not missing socio-demographic data. Diabetes was identified using ICD-9-CM diagnosis codes in the medical claims and national drug codes in the pharmacy claims (see S1 File).

### Measures

#### Outcome variables

The outcome of interest was a count of individuals’ diabetes PPHs occurring between 2011 and 2013. Diabetes PPHs were identified using the Agency for Healthcare Research and Quality’s (AHRQ) Prevention Quality Indicators v6.0 [13] logic. All diabetes-related indicators were included (i.e., diabetes long-term complications, diabetes short-term complications, uncontrolled diabetes, and lower-extremity amputation among patients with diabetes).

#### Primary explanatory variables

Behavioral health conditions documented on claims for healthcare services rendered between 2011 and 2013 were the primary explanatory variables in the study. We identified conditions by examining schizophrenia, bipolar, depression and other non-bipolar mood disorders, anxiety, adjustment disorder, alcohol use disorder, and drug use disorder ICD-9-CM diagnosis codes (see S2 File [25]). Additionally, a simple count of each person’s behavioral health conditions as identified by ICD-9-CM diagnosis codes represented behavioral health complexity (i.e., 0, 1, 2, 3, ≥4). Each type of behavioral health condition listed above was counted a maximum of one time (e.g., if a person received diagnoses for both catatonic and simple-type schizophrenia, schizophrenia is only counted once).

#### Explanatory covariates

We included variables in the statistical models to adjust for potential confounders in the relationship between behavioral health conditions and diabetes PPHs. Anderson’s Behavioral Model of Health Services Use was used to select these covariates. This model posits that health services use is affected by individuals’ predisposing characteristics (e.g., age, sex), enabling resources (e.g., distance to provider, income), and clinical need (e.g., chronic conditions, pain) [26]. Our covariates included age, sex, Census region, federal poverty levels (FPL) in the county of residence, urban-rural category, and physical health status variables based on healthcare services rendered or prescriptions filled between 2011 and 2013. Physical health status variables included asthma, chronic obstructive pulmonary disorder (COPD), chronic pain/pain-related conditions, tobacco use, renal failure, chronic heart conditions, cancer, chronic hypertension, chronic cerebrovascular disease, obesity, and chronic hyperlipidemia (see S3 File [27, 28]).

### Statistical analyses

We determined the number of people in each category of the explanatory variables, calculated the count of diabetes PPHs that each person experienced during the 3-year period, and conducted two sets of unadjusted bivariate analyses. First, we used Kruskal-Wallis tests to examine the relationships between the explanatory variables and increasing counts of diabetes PPHs. Second, we used chi-squared tests to examine the relationships between the explanatory variables and the likelihood of having one or more diabetes PPHs.

We also explored the adjusted associations between the explanatory variables and the count of diabetes PPHs using negative-binomial hurdle regression models. This type of two-part regression model accommodates the fact that two different probability functions are likely generating the zero and the positive values [29]. As such, it enables us to examine the relationship between behavioral health conditions and the likelihood of experiencing at least one PPH separately from the relationship between behavioral health conditions and increasing counts of PPHs in those with one or more PPHs.

We ran two negative-binomial hurdle regression models. Model 1 explored the relationship between individual behavioral health conditions and diabetes PPHs, controlling for sociodemographic and physical health covariates. Model 2 examined the relationship between behavioral health complexity and diabetes PPHs, again controlling for sociodemographic and physical health covariates. Additionally, because we identified no standard methodology for identifying persons with chronic pain within administrative data, a sensitivity test was conducted to determine if the relationship between behavioral health conditions and diabetes PPHs was sensitive to differing operational definitions of our chronic pain covariate.

All hypotheses were a priori, and all statistical testing was conducted in Stata 14.2, was 2-sided, and significance was tested at p<0.05.

## Results

Of the 4 million people in the available Optum Clinformatics data, 229,179 were adults ages 20 to 64 with diabetes. We excluded 140 of those individuals due to missing socio-demographic information. Thus, 229,039 individuals met inclusion criteria for the study. A total of 4,521 diabetes PPHs were experienced by 3,246 of these persons (3,246/229,039; 1.42%; 95% Confidence Interval [CI] 1.37–1.47%). In addition, 623 individuals (623/229,039; 0.27% CI 0.25%-0.29%) experienced more than one diabetes PPH. Further, 47,701 (20.83%; CI 20.66%-20.99%) of persons in the study had at least one behavioral health condition; 34.30% (16,361/47,701; CI 33.88%-34.73%) of those persons had more than one behavioral health condition.

### Relationships between diabetes PPHs and behavioral health conditions

The 20.83% of persons with one or more behavioral health conditions experienced 43.62% (1,972/4,521; CI 42.18%-45.07%) of all diabetes PPHs, and the 7.14% of persons with more than one behavioral health condition experienced 24.77% (1,120/4,521; CI 23.54%-26.05%) of all diabetes PPHs.

#### Mental health conditions

In unadjusted analyses, all mental health conditions (i.e., schizophrenia, bipolar, depression/other mood disorder, anxiety, and adjustment disorders) were associated with an increased likelihood of having at least one diabetes PPH and increased counts of diabetes PPHs (p<0.001 for all; see Table 1). However, in the adjusted analysis, only two mental health conditions, schizophrenia and depression/other mood disorder, were independently associated with an increased likelihood of having at least one diabetes PPH (p<0.001 for both; see Table 2). Similarly, depression/other mood disorder was the only mental health condition independently associated with increasing counts of PPHs in those with at least one PPH (p<0.001) although schizophrenia approached significance (p = 0.095; see Table 2).

**Table 1:** Characteristics of persons with diabetes by diabetes-related potentially preventable hospitalization (PPH) count, based on medical and pharmacy claims data with 2011–2013 service dates (n = 229,039). PPH counts are truncated at three in Table 1 for presentation purposes, but counts were not truncated in statistical analyses.

	Number in Category	# with 0 PPHs	% with 0 PPHs	# with 1 PPH	% with 1 PPH	# with 2 PPHs	% with 2 PPHs	# with ≥3 PPHs	% with ≥3 PPHs	p-value	# with ≥1 PPH	% with ≥1 PPH	p-value
**Total**	229,039	225,793	98.58%	2,603	1.14%	399	0.17%	224	0.11%	n/a	3,246	1.42%	n/a
**Gender**													
Male	123,582	121,634	98.42%	1,579	1.28%	233	0.19%	136	0.11%	<0.001	1,948	1.58%	<0.001
Female	105,457	104,159	98.77%	1,024	0.97%	166	0.16%	108	0.10%		1,298	1.23%	
**Age Group**													
20–29	6,128	5,793	94.53%	247	4.03%	47	0.77%	41	0.67%	<0.001	335	5.47%	<0.001
30–44	45,089	44,329	98.31%	608	1.35%	85	0.19%	67	0.15%		760	1.69%	
45–64	177,822	175,671	98.79%	1,748	0.98%	267	0.15%	136	0.08%		2,151	1.21%	
**Census Region**													
Midwest	56,746	55,853	98.43%	728	1.28%	104	0.18%	61	0.11%	<0.001	893	1.24%	<0.001
Northeast	54,778	54,099	98.76%	528	0.96%	90	0.16%	61	0.11%		679	1.57%	
South	50,336	49,588	98.51%	593	1.18%	99	0.20%	56	0.11%		748	1.49%	
West	67,179	66,253	98.62%	754	1.12%	106	0.16%	66	0.10%		926	1.38%	
**Federal Poverty Level (FPL) in County**													
<15% of Households Under FPL	110,889	109,394	98.65%	1,202	1.08%	179	0.16%	114	0.10%	0.010	1,495	1.35%	0.007
> = 15% of Households Under FPL	118,150	116,399	98.52%	1,401	1.19%	220	0.19%	130	0.11%		1,751	1.48%	
**Urban-Rural Category**													
Large Central Metro	62,228	61,291	98.49%	726	1.17%	134	0.22%	77	0.12%	0.009	937	1.51%	0.009
Large Fringe Metro	79,524	78,378	98.56%	925	1.16%	133	0.17%	88	0.11%		1,146	1.44%	
Medium Metro	45,829	45,194	98.61%	522	1.14%	72	0.16%	41	0.09%		635	1.39%	
Small Metro	17,067	16,826	98.59%	189	1.11%	31	0.18%	21	0.12%		241	1.41%	
Micropolitan	14,318	14,140	98.76%	153	1.07%	16	0.11%	9	0.06%		178	1.24%	
Noncore	10,073	9,964	98.92%	88	0.87%	13	0.13%	13	0.08%		109	1.08%	
**COPD**													
Condition absent	219,861	216,865	98.64%	2,429	1.10%	352	0.16%	215	0.10%	<0.001	2,996	1.36%	<0.001
Condition present	9,178	8,928	97.28$	174	1.90%	47	0.51%	29	0.32%		250	2.72%	
**Asthma**													
Condition absent	211,056	208,085	98.59%	2,389	1.13%	368	0.17%	214	0.10%	0.374	2,971	1.41%	0.186
Condition present	17,983	17,708	98.47%	214	1.19%	31	0.17%	30	0.17%		275	1.53%	
**Chronic Pain/Pain-Related Condition**													
Condition absent	167,554	165,237	98.62%	1,934	1.15%	251	0.15%	132	0.08%	0.010	2,317	1.38%	0.022
Condition present	61,485	60,556	98.49%	669	1.09%	148	0.24%	112	0.18%		929	1.51%	
**Tobacco Use**													
Condition absent	205,385	202,974	98.83%	1,992	0.97%	275	0.13%	144	0.07%	<0.001	2,411	1.17%	<0.001
Condition present	23,654	22,819	96.47%	611	2.58%	124	0.52%	100	0.42%		835	3.53%	
**Renal Failure/Chronic Kidney Disease**													
Condition absent	226,017	223,208	98.76%	2,282	1.01%	328	0.15%	199	0.09%	<0.001	2,809	1.24%	<0.001
Condition present	3,022	2,585	85.54%	321	10.62%	71	2.35%	45	1.49%		437	14.46%	
**Chronic Heart Condition**													
Condition absent	179,492	177,602	98.95%	1,620	0.90%	178	0.10%	92	0.05%	<0.001	1,890	1.05%	<0.001
Condition present	49,547	48,191	97.26%	983	1.98%	221	0.45%	152	0.31%		1,356	2.74%	
**Cancer**													
Condition absent	208,746	205,793	98.59%	2,364	1.13%	367	0.18%	222	0.11%	0.374	2,953	1.41%	0.737
Condition present	20,293	20,000	98.56%	239	1.18%	32	0.16%	22	0.11%		293	1.44%	
**Chronic Hypertension**													
Condition absent	64,213	63,501	98.89%	605	0.94%	65	0.10%	42	0.07%	<0.001	712	1.11%	<0.001
Condition present	164,826	162,292	98.46%	1,998	1.21%	334	0.20%	202	0.12%		2,534	1.54%	
**Chronic Cerebrovascular Disease**													
Condition absent	218,487	215,601	98.68%	2,343	1.07%	336	0.15%	207	0.09%	<0.001	2,886	1.32%	<0.001
Condition present	10,552	10,192	96.59%	260	2.46%	63	0.60%	37	0.35%		360	3.41%	
**Obesity**													
Condition absent	175,276	173,058	98.73%	1,790	1.02%	265	0.15%	163	0.09%	<0.001	2,218	1.27%	<0.001
Condition present	53,763	52,735	98.09%	813	1.51%	134	0.25%	81	0.15%		1,028	1.91%	
**Chronic Hyperlipidemia**													
Condition absent	56,526	55,653	98.46%	709	1.25%	99	0.18%	65	0.11%	0.002	873	1.54%	0.003
Condition present	172,513	170,140	98.62%	1,894	1.10%	300	0.17%	179	0.10%		2,373	1.38%	
**Alcohol Use Disorder**													
Condition absent	225,921	222,864	98.65%	2,468	1.09%	366	0.16%	223	0.10%	<0.001	3,057	1.35%	<0.001
Condition present	3,118	2,929	93.94%	135	4.33%	33	1.06%	21	0.67%		189	6.06%	
**Drug Use Disorder**													
Condition absent	226,645	223,574	98.65%	2,510	1.11%	358	0.16%	203	0.09%	<0.001	3,071	1.35%	<0.001
Condition present	2,394	2,219	92.69%	93	3.88%	41	1.71%	41	1.71%		175	7.31%	
**Schizophrenia**													
Condition absent	226,782	223,678	98.63%	2,513	1.11%	376	0.17%	215	0.09%	<0.001	3,104	1.37%	<0.001
Condition present	2,257	2,115	93.71%	90	3.99%	23	1.02%	29	1.28%		142	6.29%	
**Bipolar**													
Condition absent	226,004	222,860	98.61%	2,532	1.12%	387	0.17%	225	0.10%	<0.001	3,144	1.39%	<0.001
Condition present	3,035	2,933	96.64%	71	2.34%	12	0.40%	19	0.63%		102	3.36%	
**Depression & Other Mood Disorders**													
Condition absent	199,329	196,864	98.76%	2,072	1.04%	268	0.13%	125	0.06%	<0.001	2,465	1.24%	<0.001
Condition present	29,710	28,929	97.37%	531	1.79%	131	0.44%	119	0.40%		781	2.63%	
**Anxiety**													
Condition absent	206,239	203,528	98.69%	2,232	1.08%	318	0.15%	161	0.08%	<0.001	2,711	1.31%	<0.001
Condition present	22,800	22,265	97.65%	371	1.63%	81	0.36%	83	0.36%		535	2.35%	
**Adjustment Disorder**													
Condition absent	222,411	219,324	98.61%	2,495	1.12%	372	0.17%	220	0.10%	<0.001	3,087	1.39%	<0.001
Condition present	6,628	6,469	97.60%	108	1.63%	27	0.41%	24	0.36%		159	2.40%	
**BH Complexity Category**													
No BH Conditions	181,338	179,282	98.87%	1,745	0.96%	216	0.12%	95	0.05%	<0.001	2,056	1.13%	<0.001
1 BH Condition	31,340	30,719	98.02%	489	1.56%	83	0.26%	49	0.16%		621	1.98%	
2 BH Conditions	12,022	11,669	97.06%	249	2.07%	57	0.47%	47	0.39%		353	2.94%	
3 BH Conditions	3,219	3,082	95.74%	81	2.52%	26	0.81%	30	0.93%		137	4.26%	
4+ BH Conditions	1,120	1,041	92.95%	39	3.48%	17	1.52%	23	2.05%		79	7.05%	

Abbreviations

BH: Behavioral health (including both mental health conditions and substance use disorders)

COPD: Chronic obstructive pulmonary disease

FPL: Federal poverty level

PPH: Diabetes-related potentially preventable hospitalization

**Table 2:** Adjusted odds ratios (aOR) and adjusted risk ratios (aRR) of potentially preventable hospitalizations for diabetes (PPH) in individuals with and without behavioral health conditions (n = 229,039). Logit results examine findings regarding the presence or absence of any PPH, whereas the negative binomial results examine the findings regarding the increasing counts of PPHs in individuals with one or more PPH. Analyses were adjusted for sociodemographic and physical health covariates. For full Model 1 results see S4 File.

	Logit				Negative Binomial			
	aOR	95% Confidence Interval		p-value	aRR	95% Confidence Interval		p-value
**Alcohol Use Disorder**								
Condition absent	1.000				1.000			
Condition present	1.768	1.448	2.158	<0.001	0.990	0.648	1.512	0.964
**Drug Use Disorder**								
Condition absent	1.000				1.000			
Condition present	1.982	1.608	2.443	<0.001	2.238	1.563	3.202	<0.001
**Schizophrenia**								
Condition absent	1.000				1.000			
Condition present	1.611	1.294	2.005	<0.001	1.414	0.942	2.121	0.095
**Bipolar**								
Condition absent	1.000				1.000			
Condition present	0.988	0.778	1.254	0.922	1.342	0.780	2.307	0.288
**Depression & Other Mood Disorders**								
Condition absent	1.000				1.000			
Condition present	1.546	1.394	1.714	<0.001	2.007	1.563	2.576	<0.001
**Anxiety**								
Condition absent	1.000				1.000			
Condition present	1.029	0.917	1.154	0.629	1.206	0.915	1.590	0.184
**Adjustment Disorder**								
Condition absent	1.000				1.000			
Condition present	1.160	0.973	1.382	0.098	1.279	0.886	1.847	0.189

Abbreviations

aOR: Adjusted odds ratio

aRR: Adjusted risk ratio

FPL: Federal poverty level

PPH: Diabetes-related potentially preventable hospitalization

#### Substance use disorders

Substance use disorders were also associated with diabetes PPHs. In unadjusted and adjusted analyses, both alcohol use disorders and drug use disorders were associated with an increased likelihood of having at least one diabetes PPH (p<0.001 for all), and in unadjusted analyses both were associated with increasing counts of diabetes PPHs (p<0.001 for both; see Tables 1 and 2). However, in adjusted analyses, only drug use disorders were associated with an increased likelihood of increasing numbers of diabetes PPHs in persons with at least one PPH (p<0.001); this relationship was nonsignificant for alcohol use disorders (p = 0.964). Results of bivariate and multivariable analyses of individual behavioral health variables are in Tables 1 and 2, respectively, and detailed Model 1 results are in S4 File.

#### Behavioral health complexity

Increasing behavioral health complexity as measured by a count of diagnosed behavioral health conditions was associated with an increased likelihood of having one or more diabetes PPH in unadjusted analysis (p<0.001; see Table 1) and adjusted analysis (p<0.001 for all categories; see Table 3). Further, increasing behavioral health complexity was associated with increasing counts of diabetes PPHs in unadjusted (p<0.001; see Table 1) and adjusted analyses (p<0.001 to p = 0.015). See Table 3 for findings related to behavioral health complexity from Model 2, the multivariable model examining this variable, and see S4 File for detailed results of this model.

**Table 3:** Adjusted odds ratios (aOR) and adjusted risk ratios (aRR) of potentially preventable hospitalizations for diabetes (PPH) by behavioral health (BH) complexity level (n = 229,039). Logit results examine findings regarding the presence or absence of any PPH, whereas the negative binomial results examine the findings regarding the increasing counts of PPHs in individuals with one or more PPH. Behavioral health complexity represents a simple count of each person’s behavioral health conditions, including both mental health conditions and substance use disorders. Analyses were adjusted for sociodemographic and physical health covariates. For full Model 2 results see S4 File.

	Logit				Negative Binomial			
	aOR	95% Confidence Interval		p-value	aRR	95% Confidence Interval		p-value
No BH Conditions	1.000 (reference)				1.000 (reference)			
1 BH Condition	1.566	1.424	1.721	<0.001	1.389	1.067	1.809	0.015
2 BH Conditions	2.081	1.837	2.358	<0.001	2.203	1.641	2.958	<0.001
3 BH Conditions	2.536	2.096	3.069	<0.001	3.549	2.431	5.181	<0.001
> = 4 BH Conditions	3.339	2.581	4.319	<0.001	6.470	3.708	11.288	<0.001

Abbreviations

aOR: Adjusted odds ratio

aRR: Adjusted risk ratio

BH: Behavioral health (including both mental health conditions and substance use disorders)

PPH: Diabetes-related potentially preventable hospitalization

Our sensitivity analysis indicated that the findings described above were robust to variations in the operational definition of our chronic pain covariate (see S5 File).

## Discussion & conclusions

PPHs for diabetes were disproportionately concentrated in individuals with co-occurring behavioral health conditions. While 20.8% of persons with diabetes had a comorbid behavioral health condition, this group experienced 43.6% of all diabetes PPHs. These findings suggest that the outpatient care of individuals who have both diabetes and behavioral health conditions may be suboptimal, and this suboptimal care results in measurably poorer health outcomes.

However, contrary to our hypotheses, not all persons with comorbid diabetes and behavioral health conditions are at increased risk of outpatient care quality deficits as measured by diabetes PPHs; the relationship varied by condition. Depression/other mood disorder was associated with an increased likelihood of having at least one diabetes PPH and recurring PPHs in those with at least one PPH. These results align with our hypotheses and reiterate previous findings that depression increases risk of diabetes related hospitalizations and other ACSCs [9, 16]. Conversely, our adjusted analyses did not identify significant independent associations between diabetes PPHs and anxiety or adjustment disorders. These results are in contrast to those of Mai et al. [9] who found that adjustment disorders and anxiety disorders (termed “neurotic disorders” in their paper) were associated with an increased likelihood of experiencing a hospitalization for diabetes complications. The disparate findings may be due to differing methodologies in categorizing patients with multiple co-occurring behavioral health conditions. We included all behavioral health conditions in the analysis separately, whereas Mai et al. [9] assigned patients to a single behavioral health condition based on a hierarchy [30]. Patients with both anxiety and depression were deemed to have anxiety, and patients with both adjustment disorder and depression were deemed to have adjustment disorder. Thus, the aforementioned association between depression and diabetes PPHs may explain their positive results.

Additionally, we found that schizophrenia was associated with an increased likelihood of having at least one diabetes PPH, although schizophrenia was not associated with recurring PPHs over time. However, bipolar was only associated with an increased likelihood of any diabetes PPH and increased counts of diabetes PPHs in unadjusted analyses. These findings are consistent with that of Druss and colleagues [22]. They examined US Medicaid data and identified significant positive associations between schizophrenia and hospitalizations for ACSCs of all types but did not find significant relationships between ACSC hospitalizations and bipolar disorder. In contrast, our findings are inconsistent with that of Davydow and colleagues [17] who identified independent positive associations between diabetes PPHs and both schizophrenia and bipolar in a Danish sample of patients, and they are inconsistent with Mai et al. who identified no association between schizophrenia and experiencing at least one hospitalization for diabetes in an Australian sample of patients [9]. Our findings are also different from those of Leung and colleagues [21] who identified a decreased risk of diabetes PPHs in individuals with schizophrenia who were enrolled in Medicare or Medicaid in one US state. On the other hand, they too found no association between diabetes PPHs and bipolar [21]. The inconsistency in research findings regarding the relationship between diabetes PPHs and these serious mental illnesses mirror the inconsistency in the results of past research on the relationship between serious mental illness and the quality of outpatient diabetes care received [12], as would be expected given that diabetes PPHs are indicators of outpatient care quality deficits [13]. A number of factors influence the quality of care that a patient receives, including patient, delivery system, and insurance characteristics [12]. It is likely that these factors underlie the variations in findings described above; our study focused on US patients enrolled in commercial insurance plans, while the other studies examined patients from other countries [9, 17] or US patients with public insurance [21, 22].

However, findings regarding substance use disorders are uniform across studies. Consistent with past research [9, 21, 22], we found that persons with diabetes who have comorbid alcohol or drug use disorders had an increased likelihood of having ≥1 diabetes PPH. Similar findings have been observed in other populations [18]. We also expanded on past research by identifying that, while recurring diabetes PPHs were not observed in individuals with alcohol use disorders and at least one diabetes PPH, people with both diabetes and drug use disorders who experience at least one diabetes PPH have an increased risk of experiencing recurring diabetes PPHs over time. The observed relationships between substance use disorders and diabetes PPHs are likely consequences of the diabetes care quality deficits experienced by persons with co-occurring substance use disorders [7, 8, 10]. Given that persons with comorbid drug use disorders are significantly more at risk for multiple PPHs than subjects with other comorbid behavioral health conditions, future research is needed to understand how best to prevent repeat PPHs in this population. Preventing PPHs in this population presents unique challenges because repeated relapses are common, and–by definition–individuals with such disorders may neglect other areas of their life while spending a significant amount of time obtaining, using, or recovering from their substance(s) of choice [31].

Our results illustrate the importance of looking beyond individual behavioral health conditions, as over one-third of persons with comorbid diabetes and behavioral health conditions were diagnosed with multiple behavioral health conditions. Persons in this group were at even greater risk of experiencing diabetes PPHs. In total, approximately one quarter of diabetes PPHs were experienced by the 7.1% of persons with diabetes who were diagnosed with ≥1 behavioral health condition, and the risk of PPHs increased as the number of behavioral health conditions increased. These are important findings, as we identified no previous studies examining the association between increasing PPH risk and increasing numbers of behavioral health conditions. However, our results are consistent with past research that found that the likelihood of receiving recommended screenings (e.g., HbA1c, LDL-C) decreased and the likelihood of having poor lipemic or glycemic control increased as the number of behavioral health conditions increased [8]. Our results build on those findings, suggesting that these care quality deficits are likely resulting in measurably poorer outcomes for people with multiple or complex behavioral health conditions.

Enhanced outpatient care strategies that improve care quality may reduce the risk of PPHs in persons with diabetes and multiple behavioral health conditions as well as those with diabetes and either co-occurring schizophrenia, depression, or substance use disorder. An evidence-based model of care that may mitigate diabetes PPH risk in these populations is to better integrate behavioral and physical healthcare services. Mental health patients receiving integrated care that included both mental health services and physical health care generally receive a higher quality of care, including higher-quality services for prevention of complications related to diabetes [32]. There is also a need to integrate substance use screening into diabetes treatment programs for better diabetes management and substance use prevention and treatment [33]. Accordingly, current practice guidelines encourage shared responsibility between physical and behavioral health providers and recommend ongoing integrated physical and behavioral health care [34]. However, behavioral health specialists may not feel responsible for providing standard diabetes care services to their patients, and some do not have the training to manage diabetes in their patients [35]. Additionally, providers may experience reimbursement-related challenges when implementing integrated healthcare programs [36]. Hence, a multilevel cultural shift backed by payers, providers, and policymakers will be required to create a functioning and sustainable integrative care system.

The outpatient care quality deficits that likely underlie the PPHs observed in this study may also be related in part to provider attitudes and beliefs. Stigma related to mental health and fear of being labeled impedes people from seeking services for their mental health needs [37]. For persons who do seek treatment, healthcare providers may have negative attitudes towards and consequently provide a poor quality of care to patients with mental health and/or substance use disorders [38, 39]. Stigma in healthcare settings often results from lack of awareness or skills and workplace culture [38]. By addressing these issues, outpatient care quality can be improved for the vulnerable population of persons with comorbid diabetes and behavioral health conditions.

Additionally, the majority of care that patients with chronic conditions receive is self-care [40], so the importance of self-care in reducing PPH risk in the population of persons with co-occurring diabetes and behavioral health conditions cannot be understated. Behaviors such as healthy eating, blood sugar monitoring, medication adherence, and physical activity are all associated with positive diabetes outcomes [41]. Outpatient providers play a key role in encouraging self-care [41]. Given that substance abuse disorders and depression may be associated with suboptimal self-care [42, 43], outpatient providers should consider providing self-management support to persons in these groups in order to facilitate self-care [40, 44].

Furthermore, community-based diabetes self-management programs have shown promise in improving diabetes-related knowledge and health outcomes [45, 46]. For example, in a pilot test and subsequent randomized control trial of a community-based program delivered by a team of nurses and peer educators, researchers observed significant improvement in diabetes knowledge among people with diabetes and mental health disorders after 60 weeks [47, 48]. Another pilot test of a telehealth intervention integrated with nurse case management services displayed improvement in diabetes related outcomes among people with mental health conditions at 6 months [49]. However, few randomized controlled trials have examined the effectiveness of community-based diabetes self-care interventions for persons with serious mental illness [50] so more research is needed.

While our study provides important insights into the relationships between diabetes PPHs and behavioral health conditions, there are limitations. The cross sectional observational design, while consistent with other studies examining PPHs [14], disallows us from making causal statements about the relationship between diabetes PPHs and behavioral health conditions. Further, we were unable to include individuals who were 18 or 19 years of age. While the logic that we used to identify diabetes PPHs is applicable to persons 18 and older [13], our data source grouped individuals into 5-year age groups (e.g., 15 to 19 years). Additionally, while claims data provide important insights into patients’ healthcare conditions and typically reflect accurate diagnoses, only diagnoses associated with services and included on claims can be analyzed. Patients with undiagnosed conditions or with conditions not reflected in claims would not be identified as having these conditions.

Similarly, claims data only contains information relevant to payers, so we were unable to control for many socioenvironmental variables (e.g., household composition, social support, stress) that would be expected to vary greatly within the heterogeneous commercially insured population. We were also unable to control for modifiable risk factors and individual lifestyle choices that have previously been found to be associated with poor physical health in persons with behavioral health conditions (e.g., lack of exercise, poor nutrition, motivation [51]). In addition, individuals in our sample were continuously enrolled in commercial insurance plans for a three-year period, so persons without stable coverage and persons who migrate into Medicaid or other coverage are not represented by this analysis.

We provide important insights regarding the strong association between drug use disorders and experiencing multiple diabetes PPHs, but different drugs (e.g., cannabis, opioids, sedatives) may have differing associations with PPH. We did not examine non-alcohol substances separately, so additional research is warranted. Finally, behavioral health complexity measured by unique diagnosed condition counts does not reflect the range of morbidities that may be observed within persons with a single condition such as schizophrenia or substance use disorder.

While our study provides new information about the relationship between behavioral health conditions and diabetes PPHs, some questions remain unanswered. We did not investigate whether improvements in the quality of outpatient care provided to persons with behavioral health conditions mitigates their risk of diabetes PPHs. When future research is conducted to explore this topic, a number of standard quality measures could be leveraged, including Health Effectiveness Data and Information Set (HEDIS) measures (e.g., Diabetes Monitoring for People with Diabetes and Schizophrenia which examines HbA1c testing, Diabetes Care for People with Serious Mental Illness which examines HbA1c control)[52]. Further, the relationship between atypical antipsychotics, commonly used for treatment of psychotic disorders, bipolar disorder, and some forms of depression, is well known. Therefore, future studies needed to explore whether psychiatric medications or adherence to those medications increases or decreases the risk of diabetes PPHs in the populations of persons with behavioral health conditions.

We provided important insights regarding the strong association between drug use disorders and experiencing multiple diabetes PPHs, but different drugs (e.g., cannabis, opioids, sedatives) may have differing associations with PPH. We did not examine non-alcohol substances separately, so additional research is warranted. And, while our study provides new information regarding diabetes PPHs in persons with diabetes, future studies are needed to examine whether individual behavioral health conditions are each independently associated with an increased likelihood of experiencing at least one PPH or increasing numbers of PPHs in persons with other chronic ambulatory care sensitive conditions (e.g., chronic obstructive pulmonary disease, congestive heart failure, asthma).

Despite the limitations, this work provides important, actionable insights into the relationship between diabetes PPHs and behavioral health conditions. In total, 43.6% of all diabetes PPHs were experienced by the 20.8% of persons with diabetes and a comorbid behavioral health condition. Specifically, patients with depression, schizophrenia, substance use disorders, or multiple behavioral health conditions are at increased risk of experiencing at least one diabetes PPH. Patients with depression, drug use disorder, or multiple behavioral health conditions are especially at-risk, as they are susceptible to recurring diabetes PPHs over time. Given that diabetes PPHs are often preventable with timely, high-quality outpatient care, providers, policymakers and payers should implement targeted interventions and enhanced care models to improve the quality of clinical and self-care received by these vulnerable patients.

## Supporting information

S1 File Operational definition of diabetes. This file contains information about how individuals with diabetes were identified for the study.
(XLSX)

S2 File Operational definition of behavioral health conditions. This file contains detailed information regarding the operational definition of the behavioral health variables used in the statistical models.
(XLSX)

S3 File Operational definition of the physical health status variables. This file contains detailed information regarding the operational definition of the physical health status variables used in the statistical models.
(XLSX)

S4 File Model results. This file contains detailed results of the two negative binomial-logit hurdle regression models examining the relationship between behavioral health conditions and potentially preventable hospitalizations for diabetes. Model 1 includes individual behavioral health conditions. Model 2 includes a count of behavioral health conditions.
(PDF)

S5 File Sensitivity analysis. This file contains a sensitivity analysis examining the robustness of the study findings to variations in the operational definition of chronic pain-related conditions.
(PDF)

## References

[1] Centers for Disease Control and Prevention. National Diabetes Statistics Report, 2017. Atlanta, GA: US Dept of Health and Human Services, 2017.

[2] Heron MP. Deaths: Leading causes for 2016. National Vital Statistics Reports. 2018;67(6):1–76. 30248017

[3] Huang ES, Brown SE, Ewigman BG, Foley EC, Meltzer DO. Patient perceptions of quality of life with diabetes-related complications and treatments. Diabetes Care. 2007;30(10):2478–83. 10.2337/dc07-0499. 17623824 PMC2288662

[4] American Diabetes Association. Economic costs of diabetes in the U.S. in 2017. Diabetes Care. 2018;41(5):917–28. 10.2337/dci18-0007 29567642 PMC5911784

[5] Li R, Bilik D, Brown MB, Zhang P, Ettner SL, Ackermann RT, et al Medical costs associated with type 2 diabetes complications and comorbidities. Am J Manag Care. 2013;19(5):421–30. 23781894 PMC4337403

[6] Armstrong C. ADA updates standards of medical care for patients with diabetes mellitus. American family physician. 2017;95(1):40–3. 28075100

[7] Clark RE, Weir S, Ouellette RA, Zhang J, Baxter JD. Beyond health plans: behavioral health disorders and quality of diabetes and asthma care for Medicaid beneficiaries. Med Care. 2009;47(5):545–52. 10.1097/MLR.0b013e318190db45 19319000

[8] Frayne SM, Halanych JH, Miller DR, Wang F, Lin H, Pogach L, et al Disparities in diabetes care: impact of mental illness. Arch Intern Med. 2005;165(22):2631–8. 10.1001/archinte.165.22.2631 16344421

[9] Mai Q, Holman CD, Sanfilippo FM, Emery JD, Preen DB. Mental illness related disparities in diabetes prevalence, quality of care and outcomes: a population-based longitudinal study. BMC Med. 2011;9:118 10.1186/1741-7015-9-118 22044777 PMC3215928

[10] Desai MM, Rosenheck RA, Druss BG, Perlin JB. Mental disorders and quality of diabetes care in the veterans health administration. Am J Psychiatry. 2002;159(9):1584–90. 10.1176/appi.ajp.159.9.1584 12202281

[11] Leung GY, Zhang J, Lin WC, Clark RE. Behavioral health disorders and adherence to measures of diabetes care quality. Am J Manag Care. 2011;17(2):144–50. 21473663

[12] McGinty EE, Baller J, Azrin ST, Juliano-Bult D, Daumit GL. Quality of medical care for persons with serious mental illness: A comprehensive review. Schizophrenia research. 2015;165(2–3):227–35. 10.1016/j.schres.2015.04.010 25936686 PMC4670551

[13] Agency for Healthcare Research and Quality. Prevention Quality Indicators Overview & Prevention Quality Indicators Resources 2017 [cited 13 January 2019]. Available from: http://www.qualityindicators.ahrq.gov/modules/pqi_resources.aspx.

[14] Cahoon EK, McGinty EE, Ford DE, Daumit GL. Schizophrenia and potentially preventable hospitalizations in the United States: a retrospective cross-sectional study. BMC psychiatry. 2013;13:37 10.1186/1471-244X-13-37 23351438 PMC3599909

[15] Lin HC, Huang CC, Chen SF, Chen YH. Increased risk of avoidable hospitalization among patients with schizophrenia. Canadian Journal of Psychiatry. 2011;56(3):171–8. 10.1177/070674371105600307 21443824

[16] Davydow DS, Fenger-Gron M, Ribe AR, Pedersen HS, Prior A, Vedsted P, et al Depression and risk of hospitalisations and rehospitalisations for ambulatory care-sensitive conditions in Denmark: a population-based cohort study. BMJ Open. 2015;5(12):e009878 10.1136/bmjopen-2015-009878 26634401 PMC4679902

[17] Davydow DS, Ribe AR, Pedersen HS, Fenger-Gron M, Cerimele JM, Vedsted P, et al Serious mental illness and risk for hospitalizations and rehospitalizations for ambulatory care-sensitive conditions in Denmark: a nationwide population-based cohort study. Med Care. 2016;54(1):90–7. 10.1097/MLR.0000000000000448 26492210

[18] Leung KS, Parks J, Topolski J. Preventable hospitalizations among adult Medicaid beneficiaries with concurrent substance use disorders. Preventive medicine reports. 2015;2:379–84. 10.1016/j.pmedr.2015.04.022 26844094 PMC4721431

[19] Kisely S, Ehrlich C, Kendall E, Lawrence D. Using avoidable admissions to measure quality of care for cardiometabolic and other physical comorbidities of psychiatric disorders: a population-based, record-linkage analysis. Canadian Journal of Psychiatry. 2015;60(11):497–506. 10.1177/070674371506001105 26720507 PMC4679130

[20] Yoon J, Yano EM, Altman L, Cordasco KM, Stockdale SE, Chow A, et al Reducing costs of acute care for ambulatory care-sensitive medical conditions: the central roles of comorbid mental illness. Med Care. 2012;50(8):705–13. 10.1097/MLR.0b013e31824e3379 22437618

[21] Leung G, Zhang J, Lin WC, Clark RE. Behavioral disorders and diabetes-related outcomes among Massachusetts Medicare and Medicaid beneficiaries. Psychiatr Serv. 2011;62(6):659–65. 10.1176/ps.62.6.pss6206_0659 21632736

[22] Druss BG, Zhao L, Cummings JR, Shim RS, Rust GS, Marcus SC. Mental comorbidity and quality of diabetes care under Medicaid: a 50-state analysis. Med Care. 2012;50(5):428–33. 10.1097/MLR.0b013e318245a528 22228248 PMC3360811

[23] Stark Casagrande S, Cowie CC. Health insurance coverage among people with and without diabetes in the U.S. adult population. Diabetes Care. 2012;35(11):2243–9. 10.2337/dc12-0257 22787175 PMC3476921

[24] Optum. Clinformatics Data Mart 2014 [cited 13 January 2019]. Available from: https://www.optum.com/content/dam/optum/resources/productSheets/Clinformatics_for_Data_Mart.pdf.

[25] Agency for Healthcare Research and Quality. Healthcare Cost and Utilization Project (HCUP): Clinical Classifications Software (CCS) for ICD-9-CM 2017 [cited 13 January 2019]. Available from: www.hcup-us.ahrq.gov/toolssoftware/ccs/ccs.jsp.

[26] Aday LA, Andersen R. A framework for the study of access to medical care. Health Services Research. 1974;9(3):208–20. 4436074 PMC1071804

[27] Agency for Healthcare Research and Quality. MEPS HC-146: 2011 Medical Conditions File 2013 [cited 13 January 2019]. Available from: http://meps.ahrq.gov/mepsweb/data_stats/download_data/pufs/h146/h146doc.shtml.

[28] Agency for Healthcare Research and Quality. Healthcare Cost and Utilization Project (HCUP): Chronic Condition Indicator (CCI) for ICD-9-CM 2016 [cited 13 January 2019]. Available from: https://www.hcup-us.ahrq.gov/toolssoftware/chronic/chronic.jsp.

[29] Jones AM. Applied econometrics for health economists: a practical guide. Abingdon: Radcliffe; 2007.

[30] Lawrence D, Holman CD, Jablensky AV, Threlfall TJ, Fuller SA. Excess cancer mortality in Western Australian psychiatric patients due to higher case fatality rates. Acta Psychiatr Scand. 2000;101(5):382–8. 10823298 10.1034/j.1600-0447.2000.101005382.x

[31] American Psychiatric Association. Diagnostic and Statistical Manual of Mental Disorders, Fifth Edition (DSM-5). Arlington, VA: American Psychiatric Association; 2013.

[32] Kilbourne AM, Pirraglia PA, Lai Z, Bauer MS, Charns MP, Greenwald D, et al Quality of general medical care among patients with serious mental illness: does colocation of services matter? Psychiatr Serv. 2011;62(8):922–8. 10.1176/ps.62.8.pss6208_0922 21807832

[33] Ghitza UE, Wu LT, Tai B. Integrating substance abuse care with community diabetes care: implications for research and clinical practice. Substance abuse and rehabilitation. 2013;4:3–10. 10.2147/SAR.S39982 23378792 PMC3558925

[34] World Health Organization. Mental health action plan 2013–2020: 66th World Health Assembly; 2013 [cited 13 January 2019]. Available from: http://www.who.int/mental_health/publications/action_plan/en/.

[35] McBain H, Lamontagne-Godwin F, Haddad M, Simpson A, Chapman J, Jones J, et al Management of type 2 diabetes mellitus in people with severe mental illness: an online cross-sectional survey of healthcare professionals. BMJ Open. 2018;8(2):e019400 10.1136/bmjopen-2017-019400 29449295 PMC5829882

[36] Kathol RG, Butler M, McAlpine DD, Kane RL. Barriers to physical and mental condition integrated service delivery. Psychosom Med. 2010;72(6):511–8. 10.1097/PSY.0b013e3181e2c4a0 20498293

[37] Corrigan P. How stigma interferes with mental health care. American Psychologist. 2004;59(7):614–25. 10.1037/0003-066X.59.7.614 15491256

[38] Knaak S, Mantler E, Szeto A. Mental illness-related stigma in healthcare: Barriers to access and care and evidence-based solutions. Healthcare Management Forum. 2017;30(2):111–6. 10.1177/0840470416679413 28929889 PMC5347358

[39] van Boekel LC, Brouwers EP, van Weeghel J, Garretsen HF. Stigma among health professionals towards patients with substance use disorders and its consequences for healthcare delivery: systematic review. Drug Alcohol Depend. 2013;131(1–2):23–35. 10.1016/j.drugalcdep.2013.02.018 23490450

[40] Greaves CJ, Campbell JL. Supporting self-care in general practice. The British Journal of General Practice. 2007;57(543):814–21. 17925140 PMC2151815

[41] Shrivastava SR, Shrivastava PS, Ramasamy J. Role of self-care in management of diabetes mellitus. Journal of diabetes and metabolic disorders. 2013;12(1):14 10.1186/2251-6581-12-14 23497559 PMC3599009

[42] Mulligan K, McBain H, Lamontagne-Godwin F, Chapman J, Flood C, Haddad M, et al Barriers to effective diabetes management—a survey of people with severe mental illness. BMC psychiatry. 2018;18(1):165 10.1186/s12888-018-1744-5 29859061 PMC5984777

[43] Mulligan K, McBain H, Lamontagne-Godwin F, Chapman J, Haddad M, Jones J, et al Barriers and enablers of type 2 diabetes self-management in people with severe mental illness. Health expectations. 2017;20(5):1020–30. 10.1111/hex.12543 28306182 PMC5600230

[44] Agency for Healthcare Research and Quality. Self-Management Support. Rockville, MD: Agency for Healthcare Research and Quality; 2014 [updated June 2014; cited 13 January 2019]. Available from: https://www.ahrq.gov/professionals/prevention-chronic-care/improve/self-mgmt/self/index.html.

[45] Freeman K, Hanlon M, Denslow S, Hooper V. Patient engagement in type 2 diabetes: a collaborative community health initiative. The Diabetes Educator. 2018;44(4):395–404. 10.1177/0145721718784262 29972097

[46] Carrasquillo O, Lebron C, Alonzo Y, Li H, Chang A, Kenya S. Effect of a community health worker intervention among Latinos with poorly controlled type 2 diabetes: The Miami Healthy Heart Initiative Randomized Clinical Trial. JAMA Intern Med. 2017;177(7):948–54. 10.1001/jamainternmed.2017.0926 28459925 PMC5818804

[47] Sajatovic M, Gunzler DD, Kanuch SW, Cassidy KA, Tatsuoka C, McCormick R, et al A 60-week prospective RCT of a self-management intervention for individuals with serious mental illness and diabetes mellitus. Psychiatr Serv. 2017;68(9):883–90. 10.1176/appi.ps.201600377 28502243 PMC5675044

[48] Sajatovic M, Dawson NV, Perzynski AT, Blixen CE, Bialko CS, McKibbin CL, et al Best practices: Optimizing care for people with serious mental illness and comorbid diabetes. Psychiatr Serv. 2011;62(9):1001–3. 10.1176/appi.ps.62.9.1001 21885575 PMC4497574

[49] Pratt SI, Bartels SJ, Mueser KT, Naslund JA, Wolfe R, Pixley HS, et al Feasibility and effectiveness of an automated telehealth intervention to improve illness self-management in people with serious psychiatric and medical disorders. Psychiatric rehabilitation journal. 2013;36(4):297–305. 10.1037/prj0000022 24320837 PMC5472060

[50] McBain H, Mulligan K, Haddad M, Flood C, Jones J, Simpson A. Self management interventions for type 2 diabetes in adult people with severe mental illness. Cochrane Database Syst Rev. 2016;4:Cd011361 10.1002/14651858.CD011361.pub2 27120555 PMC10201333

[51] Parks J, Dale S, Singer P, Foti ME. Morbidity and mortality in people with serious mental illness: technical report 13 of the National Association of State Mental Health Program Directors Medical Directors Council 2006 [cited 13 January 2019]. Available from: http://nasmhpd.org/sites/default/files/Mortality%20and%20Morbidity%20Final%20Report%208.18.08.pdf.

[52] National Committee for Quality Assurance (NCQA). HEDIS & Performance Measures 2016 [cited 13 January 2019]. Available from: http://www.ncqa.org/hedis-quality-measurement.

