# Supplementary material for: Behavioral health conditions and potentially preventable diabetes-related hospitalizations in the United States: Findings from a national sample of commercial claims data
Source: PLoS One. 2019 Feb 28;14(2):e0212955. doi: 10.1371/journal.pone.0212955 (PMC6394977; doi:10.1371/journal.pone.0212955)
Supplement: S4 File — This file contains detailed results of the two negative binomial-logit hurdle regression models examining the relationship between behavioral health conditions and potentially preventable hospitalizations for diabetes. Model 1 includes individual behavioral health conditions. Model 2 includes a count of behavioral health conditions. (PDF) [file pone.0212955.s004.pdf]

<u>Supplemental File S4</u>

Below are detailed results of two negative binomial-logit hurdle regression models examining the relationship between behavioral health conditions and potentially preventable hospitalizations for diabetes. Model 1 includes individual behavioral health conditions. Model 2 includes a count of behavioral health diagnoses.

A review of the findings for non-behavioral health variables follows the tables. The results related to the behavioral health variables are reviewed in the text of the manuscript.

<u>Model 1</u>: Adjusted odds ratios (aOR) and adjusted risk ratios (aRR) of potentially preventable hospitalizations for diabetes (PPH). Logit results examine findings regarding the presence or absence of any PPH, whereas the negative binomial results examine the findings regarding the increasing counts of PPHs in individuals with one or more PPH. The findings in this table are from Model 1, in which behavioral health variables are entered into the model individually.

	**Logit**				**Negative Binomial**			
	**aOR**	**95% Confidence Interval**		**p-value**	**aRR**	**95% Confidence Interval**		**p-value**
**Gender**								
Male	1.000				1.000			
Female	0.775	0.718	0.838	<0.001	1.102	0.892	1.362	0.367
**Age Group**								
20-29	1.000				1.000			
30-44	0.256	0.223	0.294	<0.001	0.679	0.474	0.973	0.035
45-64	0.151	0.131	0.173	<0.001	0.387	0.276	0.541	<0.001
**Census Region**								
Midwest	1.000				1.000			
Northeast	0.828	0.746	0.919	<0.001	1.607	1.207	2.138	0.001
South	0.943	0.851	1.045	0.260	1.269	0.950	1.694	0.106
West	0.868	0.787	0.957	0.005	1.060	0.803	1.401	0.679
**Federal Poverty Level (FPL) in County**								
<15% of Households Under FPL	1.000				1.000			
>=15% of Households Under FPL	1.136	1.037	1.244	0.006	1.079	0.840	1.386	0.552
**Urban-Rural Category**								
Large Central Metro	1.000				1.000			

	Logit				Negative Binomial			
	**aOR**	**95% Confidence Interval**		**p-value**	**aRR**	**95% Confidence Interval**		**p-value**
Large Fringe Metro	1.031	0.924	1.150	0.588	0.731	0.547	0.976	0.034
Medium Metro	0.935	0.840	1.041	0.222	0.634	0.465	0.864	0.004
Small Metro	0.927	0.799	1.076	0.320	0.805	0.551	1.176	0.262
Micropolitan	0.800	0.677	0.946	0.009	0.474	0.299	0.751	0.001
Noncore	0.722	0.589	0.885	0.002	0.668	0.395	1.128	0.131
**COPD**								
No Diagnosis	1.000				1.000			
Had Diagnosis	0.940	0.808	1.095	0.427	1.241	0.836	1.843	0.284
**Asthma**								
No Diagnosis	1.000				1.000			
Had Diagnosis	0.868	0.759	0.993	0.039	0.940	0.680	1.298	0.706
**Chronic Pain/Pain-Related Condition**								
No Diagnosis	1.000				1.000			
Had Diagnosis	0.874	0.803	0.951	0.002	1.559	1.241	1.959	<0.001
**Tobacco Use**								
No Diagnosis or Prescription	1.000				1.000			
Had Diagnosis or Prescription	2.173	1.974	2.391	<0.001	1.475	1.179	1.844	0.001
**Renal Failure/Chronic Kidney Disease**								
No Diagnosis	1.000				1.000			
Had Diagnosis	8.918	7.883	10.090	<0.001	1.228	0.920	1.638	0.164
**Chronic Heart Condition**								
No Diagnosis	1.000				1.000			
Had Diagnosis	1.967	1.807	2.140	<0.001	2.264	1.815	2.823	<0.001
**Cancer**								
No Diagnosis	1.000				1.000			
Had Diagnosis	0.849	0.747	0.965	0.012	0.791	0.556	1.125	0.193
**Chronic Hypertension**								
No Diagnosis	1.000				1.000			
Had Diagnosis	1.325	1.204	1.457	<0.001	1.660	1.220	2.260	0.001
**Chronic Cerebrovascular Disease**								
No Diagnosis	1.000				1.000			
Had Diagnosis	1.465	1.291	1.663	<0.001	1.031	0.749	1.418	0.852

	Logit				Negative Binomial			
	aOR	95% Confidence Interval		p-value	aRR	95% Confidence Interval		p-value
**Obesity**								
No Diagnosis	1.000				1.000			
Had Diagnosis	1.203	1.109	1.304	<0.001	0.870	0.697	1.085	0.217
**Chronic Hyperlipidemia**								
No Diagnosis	1.000				1.000			
Had Diagnosis	0.855	0.782	0.935	0.001	0.870	0.679	1.116	0.274
**Alcohol Use Disorder**								
No Diagnosis	1.000				1.000			
Had Diagnosis	1.768	1.448	2.158	<0.001	0.990	0.648	1.512	0.964
**Drug Use Disorder**								
No Diagnosis	1.000				1.000			
Had Diagnosis	1.982	1.608	2.443	<0.001	2.238	1.563	3.202	<0.001
**Schizophrenia**								
No Diagnosis	1.000				1.000			
Had Diagnosis	1.611	1.294	2.005	<0.001	1.414	0.942	2.121	0.095
**Bipolar**								
No Diagnosis	1.000				1.000			
Had Diagnosis	0.988	0.778	1.254	0.922	1.342	0.780	2.307	0.288
**Depression & Other Mood Disorders**								
No Diagnosis	1.000				1.000			
Had Diagnosis	1.546	1.394	1.714	<0.001	2.007	1.563	2.576	<0.001
**Anxiety**								
No Diagnosis	1.000				1.000			
Had Diagnosis	1.029	0.917	1.154	0.629	1.206	0.915	1.590	0.184
**Adjustment Disorder**								
No Diagnosis	1.000				1.000			
Had Diagnosis	1.160	0.973	1.382	0.098	1.279	0.886	1.847	0.189

Abbreviations:
aOR: Adjusted odds ratio
aRR: Adjusted risk ratio
COPD: Chronic obstructive pulmonary disease
FPL: Federal poverty level
PPH: Diabetes-related potentially preventable hospitalization

Model 2: Adjusted odds ratios (aOR) and adjusted risk ratios (aRR) of potentially preventable hospitalizations for diabetes (PPH) from the negative binomial-logit hurdle model containing behavioral health (BH) complexity level. Logit results examine findings regarding the presence or absence of any PPH, whereas the negative binomial results examine the findings regarding the increasing counts of PPHs in individuals with one or more PPH. Behavioral health complexity represents a simple count of each person's behavioral health conditions, including both mental health conditions and substance use disorders.

	**Logit**				**Negative Binomial**			
	**aOR**	**95% Confidence Interval**		**p-value**	**aRR**	**95% Confidence Interval**		**p-value**
**Gender**								
Male	1.000 (reference)				1.000 (reference)			
Female	0.747	0.692	0.806	<0.001	1.119	0.911	1.375	0.285
**Age Group**								
20-29	1.000 (reference)				1.000 (reference)			
30-44	0.252	0.220	0.290	<0.001	0.650	0.454	0.930	0.018
45-64	0.151	0.132	0.172	<0.001	0.372	0.266	0.519	<0.001
**Census Region**								
Midwest	1.000 (reference)				1.000 (reference)			
Northeast	0.830	0.748	0.921	<0.001	1.604	1.205	2.134	0.001
South	0.950	0.858	1.052	0.327	1.303	0.975	1.741	0.073
West	0.871	0.790	0.961	0.006	1.073	0.815	1.414	0.615
**Federal Poverty Level (FPL) in County**								
<15% of Households Under FPL	1.000 (reference)				1.000 (reference)			
>=15% of Households Under FPL	1.136	1.037	1.244	0.006	1.086	0.847	1.393	0.515
**Urban-Rural Category**								
Large Central Metro	1.000 (reference)				1.000 (reference)			
Large Fringe Metro	1.026	0.920	1.144	0.645	0.729	0.547	0.971	0.031
Medium Metro	0.923	0.830	1.028	0.144	0.632	0.465	0.861	0.004
Small Metro	0.914	0.788	1.060	0.235	0.806	0.551	1.179	0.266
Micropolitan	0.792	0.671	0.936	0.006	0.504	0.319	0.797	0.003
Noncore	0.710	0.579	0.871	0.001	0.670	0.387	1.160	0.153

	Logit				Negative Binomial			
	aOR	95% Confidence Interval		p-value	aRR	95% Confidence Interval		p-value
**COPD**								
No Diagnosis	1.000 (reference)				1.000 (reference)			
Had Diagnosis	0.942	0.810	1.096	0.440	1.236	0.835	1.829	0.290
**Asthma**								
No Diagnosis	1.000 (reference)				1.000 (reference)			
Had Diagnosis	0.855	0.748	0.977	0.021	0.925	0.672	1.274	0.634
**Chronic Pain/Pain-Related Condition**								
No Diagnosis	1.000 (reference)				1.000 (reference)			
Had Diagnosis	0.867	0.797	0.942	0.001	1.581	1.261	1.982	<0.001
**Tobacco Use**								
No Diagnosis or Prescription	1.000 (reference)				1.000 (reference)			
Had Diagnosis or Prescription	2.229	2.030	2.447	<0.001	1.541	1.239	1.918	<0.001
**Renal Failure/Chronic Kidney Disease**								
No Diagnosis	1.000 (reference)				1.000 (reference)			
Had Diagnosis	8.986	7.948	10.160	<0.001	1.235	0.930	1.638	0.144
**Chronic Heart Condition**								
No Diagnosis	1.000 (reference)				1.000 (reference)			
Had Diagnosis	1.959	1.801	2.131	<0.001	2.311	1.855	2.880	<0.001
**Cancer**								
No Diagnosis	1.000 (reference)				1.000 (reference)			
Had Diagnosis	0.846	0.745	0.960	0.010	0.785	0.548	1.125	0.187
**Chronic Hypertension**								
No Diagnosis	1.000 (reference)				1.000 (reference)			
Had Diagnosis	1.333	1.212	1.466	<0.001	1.601	1.177	2.176	0.003
**Chronic Cerebrovascular Disease**								
No Diagnosis	1.000 (reference)				1.000 (reference)			
Had Diagnosis	1.482	1.308	1.679	<0.001	1.019	0.748	1.387	0.906
**Obesity**								
No Diagnosis	1.000 (reference)				1.000 (reference)			
Had Diagnosis	1.185	1.093	1.285	<0.001	0.880	0.705	1.099	0.260
**Chronic Hyperlipidemia**								
No Diagnosis	1.000 (reference)				1.000 (reference)			

	Logit				Negative Binomial			
	aOR	95% Confidence Interval		p-value	aRR	95% Confidence Interval		p-value
Had Diagnosis	0.843	0.771	0.922	<0.001	0.910	0.711	1.164	0.454
**Behavioral Health Complexity Level**								
No BH Diagnoses	1.000 (reference)				1.000 (reference)			
1 BH Diagnosis	1.566	1.424	1.721	<0.001	1.389	1.067	1.809	0.015
2 BH Diagnoses	2.081	1.837	2.358	<0.001	2.203	1.641	2.958	<0.001
3 BH Diagnoses	2.536	2.096	3.069	<0.001	3.549	2.431	5.181	<0.001
>=4 BH Diagnoses	3.339	2.581	4.319	<0.001	6.470	3.708	11.288	<0.001

Abbreviations:
aOR: Adjusted odds ratio
aRR: Adjusted risk ratio
COPD: Chronic obstructive pulmonary disease
FPL: Federal poverty level
PPH: Diabetes-related potentially preventable hospitalization

Relationships between Diabetes PPHs and Sociodemographic Covariates

In both adjusted and unadjusted analyses, males were more likely than females to experience at least one diabetes PPH (p<0.001 for both). In unadjusted analyses males were also more likely to experience higher counts of diabetes PPHs; however, in adjusted analyses there was no gender difference in the risk of increasing counts of diabetes PPHs in persons with at least one diabetes PPH (Model 1 p=0.367; Model 2 p=0.515). In all analyses, younger age was associated with both an increased likelihood of having at least one diabetes PPH and increased counts of diabetes PPHs (p<0.001 to p=0.035). Region was also associated with diabetes PPHs in both unadjusted and adjusted analyses (p<0.001). See Supplemental File S4 for detailed results.

In unadjusted and adjusted analyses, county-level poverty was associated with an increased likelihood of having at least one diabetes PPH (p=0.007 and p=0.006, respectively). County-level poverty was also associated with increasing counts of diabetes PPHs in unadjusted analyses (p=0.010) but this relationship was not significant in adjusted analyses (Model 1 p=0.552; Model 2 p=0.515). The risk of diabetes PPHs also varied by urban-rural category in both sets of analyses. Most notably, in adjusted analyses persons in micropolitan and non-core (i.e., rural) areas were less likely to have any diabetes PPHs, and those with at least one diabetes PPH in micropolitan areas were less likely to have increasing counts of PPHs (see Supplemental File S4).

Relationships between Diabetes PPHs and Physical Health Status Covariates

In unadjusted and adjusted analyses, chronic pain, tobacco use, chronic heart conditions, and chronic hypertension were associated with the likelihood of having at least one diabetes PPH and with increasing counts of diabetes PPHs (p<0.05 for all). In addition, in unadjusted analyses chronic renal failure, chronic cerebrovascular disease, and obesity were associated with

increasing counts of PPHs. They were also associated with an increased likelihood of having at least one diabetes PPH in both unadjusted and adjusted analyses ($p<0.05$ for all). On the other hand, these three conditions were not significantly associated with increasing counts of diabetes PPHs in those with at least one diabetes PPH in adjusted analyses ($p\geq0.05$ for all).

In unadjusted analyses, neither asthma nor cancer were associated with an increased likelihood of having at least one diabetes PPH ($p=0.186$ and $p=0.737$, respectively), nor were they associated with increasing counts of diabetes PPHs in unadjusted ($p=0.374$ for both) or adjusted ($p\geq0.05$ for all) analyses. Conversely, in adjusted analyses, the relationships between these conditions and having at least one diabetes PPH were significant ($p<0.05$ for all). Further, in unadjusted analyses chronic hyperlipidemia had a protective effect, significantly reducing the likelihood of having any or increasing counts of diabetes PPHs ($p<0.05$ for all), but in adjusted analyses only the relationship between chronic hyperlipidemia and reduced odds of having at least one PPH.
