# Supplementary material for: Behavioral health conditions and potentially preventable diabetes-related hospitalizations in the United States: Findings from a national sample of commercial claims data
Source: PLoS One. 2019 Feb 28;14(2):e0212955. doi: 10.1371/journal.pone.0212955 (PMC6394977; doi:10.1371/journal.pone.0212955)
Supplement: S5 File — This file contains a sensitivity analysis examining the robustness of the study findings to variations in the operational definition of chronic pain-related conditions. (PDF) [file pone.0212955.s005.pdf]

Supplemental File S5: Sensitivity Analysis

Summary of Findings: The observed relationships between diabetes potentially preventable hospitalizations and behavioral health conditions in our primary analyses were robust to variations in the operational definition of our chronic pain covariate.

Background: There is currently no standard method to apply to claims data in order to identify persons with chronic pain. As a result, we tested whether our conclusions regarding the relationship between diabetes potentially preventable hospitalizations (PPHs) and behavioral health conditions changed if our operational definition of pain changed.

Primary vs. Alternative Definitions of Chronic Pain: In both our primary analyses and our sensitivity analyses, persons with chronic pain were identified based ICD-9 diagnosis codes listed in Supplemental File S5. In both cases, the diagnosis must have persisted over time; however, the specific requirements differed:

- Definition in primary analyses: One or more of these diagnoses had to be found on claims for at least three different dates of service, and the first and last must be >=90 days apart.
- Definition in sensitivity analyses: One or more of these diagnoses had to be found on claims for at least two different dates of service, and they must be >=90 days apart.

Results: In the primary analysis, 26.8% of our analytic sample were identified as having chronic pain, while 31.1% had chronic pain under the alternative definition. For both definitions, chronic pain was significantly associated with the likelihood of having one or more diabetes-related potentially preventable hospitalization (see tables S5a and S5b on the following page).

Table S5a: Univariate and bivariate analyses of chronic pain as operationalized in the primary analyses.

	Number in Category	# with 0 PPHs	% with 0 PPHs	# with 1 PPH	% with 1 PPH	# with 2 PPHs	% with 2 PPHs	# with ≥3 PPHs	% with ≥3 PPHs	p-value	# with ≥1 PPH	% with ≥1 PPH	p-value
No Chronic Pain	167,554	165,237	98.62%	1,934	1.15%	251	0.15%	132	0.08%	0.010	2,317	1.38%	0.022
Had Chronic Pain	61,485	60,556	98.49%	669	1.09%	148	0.24%	112	0.18%		929	1.51%	

Table S5b: Univariate and bivariate analyses of chronic pain when applying the alternative operational definition of pain described on the previous page.

	Number in Category	# with 0 PPHs	% with 0 PPHs	# with 1 PPH	% with 1 PPH	# with 2 PPHs	% with 2 PPHs	# with ≥3 PPHs	% with ≥3 PPHs	p-value	# with ≥1 PPH	% with ≥1 PPH	p-value
No Chronic Pain	157,895	155,721	98.62%	1,817	1.15%	251	0.15%	235	0.08%	0.023	2,174	1.38%	0.015
Had Chronic Pain	71,144	70,072	98.49%	786	1.10%	164	0.23%	122	0.17%		1072	1.51%	

In addition, the alternative definition of chronic pain did not alter our findings regarding the relationship between diabetes PPHs and our behavioral health variables as measured by Model 1 wherein individual behavioral health variables were included. The relationship between diabetes PPHs and our covariates, including the chronic pain covariate, were also not affected by the change. See Table S5c on the following pages as compared to Table 2 in the manuscript and Supplemental File S

Conclusions: There is a remarkable degree of similarity between our results when the original versus the alternative definitions of chronic pain were used in analyses. Given these findings, we determined that the observed relationships between diabetes potentially preventable hospitalizations and behavioral health conditions in our primary analyses were robust to variations in the operational definition of our chronic pain covariate.

Table S5c: Results of a negative-binomial hurdle regression model exploring the relationship between individual behavioral health conditions and diabetes PPHs, while controlling for sociodemographic and physical health status covariates. This analysis used the alternative operational definition of the chronic pain covariate.

	Logit Results				Negative Binomial Results			
	Odds Ratio	95% Confidence Interval		p-value	Risk Ratio	95% Confidence Interval		p-value
**Gender**								
Male	1.000 (reference)				1.000 (reference)			
Female	0.775	0.717	0.837	<0.001	1.106	0.895	1.367	0.350
**Age Group**								
20-29	1.000 (reference)				1.000 (reference)			
30-44	0.256	0.223	0.294	<0.001	0.691	0.483	0.990	0.044
45-64	0.150	0.131	0.172	<0.001	0.393	0.280	0.550	<0.001
**Census Region**								
Midwest	1.000 (reference)				1.000 (reference)			
Northeast	0.829	0.746	0.920	<0.001	1.604	1.205	2.136	0.001
South	0.943	0.851	1.044	0.258	1.262	0.946	1.683	0.113
West	0.868	0.787	0.958	0.005	1.068	0.807	1.413	0.647
**Federal Poverty Level (FPL) in County**								
<15% of Households Under FPL	1.000 (reference)				1.000 (reference)			
>=15% of Households Under FPL	1.136	1.036	1.244	0.006	1.084	0.843	1.394	0.529
**Urban-Rural Category**								
Large Central Metro	1.000 (reference)				1.000 (reference)			

	Logit Results				Negative Binomial Results			
	Odds Ratio	95% Confidence Interval		p-value	Risk Ratio	95% Confidence Interval		p-value
Large Fringe Metro	1.030	0.923	1.149	0.595	0.738	0.552	0.986	0.040
Medium Metro	0.935	0.840	1.041	0.219	0.638	0.468	0.870	0.004
Small Metro	0.926	0.798	1.075	0.311	0.813	0.556	1.188	0.285
Micropolitan	0.800	0.677	0.945	0.009	0.477	0.302	0.754	0.002
Noncore	0.721	0.588	0.884	0.002	0.673	0.397	1.143	0.143
**COPD**								
No Diagnosis	1.000 (reference)				1.000 (reference)			
Had Diagnosis	0.939	0.806	1.093	0.415	1.238	0.834	1.837	0.289
**Asthma**								
No Diagnosis	1.000 (reference)				1.000 (reference)			
Had Diagnosis	0.866	0.758	0.991	0.036	0.948	0.687	1.308	0.745
**Pain**								
No Diagnosis	1.000 (reference)				1.000 (reference)			
Had Diagnosis	0.896	0.827	0.972	0.008	1.454	1.164	1.815	0.001
**Tobacco Use**								
No Dx or Rx	1.000 (reference)				1.000 (reference)			
Had Dx or Rx	2.171	1.972	2.389	<0.001	1.478	1.181	1.849	0.001
**Renal Failure/Chronic Kidney Disease**								
No Diagnosis	1.000 (reference)				1.000 (reference)			
Had Diagnosis	8.930	7.893	10.104	<0.001	1.214	0.910	1.621	0.187

	Logit Results				Negative Binomial Results			
	Odds Ratio	95% Confidence Interval		p-value	Risk Ratio	95% Confidence Interval		p-value
**Chronic Heart Condition**								
No Diagnosis	1.000 (reference)				1.000 (reference)			
Had Diagnosis	1.964	1.805	2.138	<0.001	2.281	1.828	2.846	<0.001
**Cancer**								
No Diagnosis	1.000 (reference)				1.000 (reference)			
Had Diagnosis	0.848	0.746	0.964	0.012	0.796	0.559	1.134	0.206
**Chronic Hypertension**								
No Diagnosis	1.000 (reference)				1.000 (reference)			
Had Diagnosis	1.324	1.204	1.457	<0.001	1.662	1.218	2.266	0.001
**Chronic Cerebrovascular Disease**								
No Diagnosis	1.000 (reference)				1.000 (reference)			
Had Diagnosis	1.463	1.289	1.660	<0.001	1.044	0.758	1.438	0.790
**Obesity**								
No Diagnosis	1.000 (reference)				1.000 (reference)			
Had Diagnosis	1.201	1.108	1.302	<0.001	0.871	0.698	1.085	0.218
**Chronic Hyperlipidemia**								
No Diagnosis	1.000 (reference)				1.000 (reference)			
Had Diagnosis	0.855	0.782	0.935	0.001	0.868	0.676	1.115	0.267
**Alcohol Use Disorder**								
No Dx	1.000 (reference)				1.000 (reference)			

	Logit Results				Negative Binomial Results			
	Odds Ratio	95% Confidence Interval		p-value	Risk Ratio	95% Confidence Interval		p-value
Had Dx	1.770	1.450	2.161	<0.001	0.993	0.650	1.517	0.974
**Drug Use Disorder**								
No Dx	1.000 (reference)				1.000 (reference)			
Had Dx	1.970	1.599	2.427	<0.001	2.273	1.586	3.257	<0.001
**Schizophrenia**								
No Dx	1.000 (reference)				1.000 (reference)			
Had Dx	1.608	1.292	2.002	<0.001	1.408	0.939	2.112	0.098
**Bipolar**								
No Dx	1.000 (reference)				1.000 (reference)			
Had Dx	0.987	0.777	1.253	0.913	1.365	0.795	2.345	0.259
**Depression & Other Mood Disorders**								
No Dx	1.000 (reference)				1.000 (reference)			
Had Dx	1.542	1.390	1.710	<0.001	2.028	1.582	2.601	<0.001
**Anxiety**								
No Dx	1.000 (reference)				1.000 (reference)			
Had Dx	1.025	0.914	1.150	0.670	1.207	0.921	1.583	0.173
**Adjustment Disorder**								
No Dx	1.000 (reference)				1.000 (reference)			
Had Dx	1.160	0.973	1.383	0.098	1.253	0.871	1.804	0.225
